# New insights into leveraging PKG signalling to treat diseased hearts

**DOI:** 10.1186/2050-6511-16-S1-A17

**Published:** 2015-09-02

**Authors:** Dong ik Lee, Taishi Nakamura, David A Kass

**Affiliations:** 1 Division of Cardiology, Department of Medicine, The Johns Hopkins University, Baltimore, Maryland, USA

## 

Activation of a protein kinase G signalling pathway has been demonstrated to ameliorate a broad range of cardiac disease conditions, including ischemia and infarction, doxorubicin toxicity, protein misfolding disorders, pressure-overload, dilated heart failure, and dystrophin deficiency. The pathway's activation depends upon cGMP generated by either NO-sGC or NP-rGC signalling. Growing evidence shows that the former is a target of oxidative stress, depressing the functionality of NO-synthase, NO (by interaction with oxidant species), and sGC. We recently examined the impact of PKG1 oxidation, a disulphide modification at C42 residues in the homodimer subunits, on cardiac stress response. While this oxidation has been previously shown to provide a gain of function in resistance arterioles, in the myocardium, prevention of this oxidation in mice or myocytes expressing a C42S mutant knock-in was protective against pressure overload. The mechanism was not explained on the basis of a change in kinase activity, but rather by differential intracellular targeting likely related to protein interactions. A key target, transient receptor potential canonical-6 ion channel, which transduces hypertrophic and fibrotic signalling via the calcineurin/NFAT pathway, was more suppressed when PKG1 was not oxidized. PKG1 oxidation also appears to alter the capacity of various ways of stimulating the kinase, including PDE inhibition, and sGC or NP stimulation. This introduces PKG1 redox as a major regulator of its signalling effects, that also pathway and ways to activate it.

In a second set of studies, we have recently revealed that the highly cGMP selective - PDE9A – is expressed in human and mouse hearts, upregulated in diseased hearts, and plays a role in maladaptive hypertrophy, fibrosis, and dysfunction in hearts exposed to sustained pressure stress. Furthermore, its genetic or selective pharmacological suppression ameliorates this cardiac pathophysiology. Unlike PDE5A, PDE9A targets the NP signalling pathway, effectively bypassing suppression of the NO-sGC cascades as often occurs in diseased hearts due to oxidative stress. PDE9A inhibition remains effective in vivo and in vitro even if NOS is blocked. These studies reveal two novel approaches to better leverage PKG1 activation in the diseased heart, suppression of oxidative modifications of the kinase at C42, and blocking PDE9A to circumvent declines in NO-sGC signalling.

